# PP45 Core Outcome Sets For Research And Core Outcome Sets For Routine Care: Do They Overlap?

**DOI:** 10.1017/S0266462324002162

**Published:** 2025-01-07

**Authors:** Anna Kearney, Sarah Gorst, Paula Williamson, Susanna Dodd

## Abstract

**Introduction:**

Core outcome sets (COS) are increasingly being developed for routine care, but it is unclear how they compare to COS for research for a given condition, particularly regarding recommended outcomes. If similar, embedding COS within clinical practice creates opportunities for improving data for real-world evidence. This work aims to compare outcomes in COS for different settings.

**Methods:**

Cancer, neurology, cardiovascular, rheumatology, and orthopedic COS within the Core Outcomes Measures in Effectiveness Trials (COMET) database were reviewed to create matched sets of COS (COS that were developed for the same condition but different settings). Recommended outcomes were extracted along with information on COS scope (condition, population, and intervention), patient involvement, and year of publication. Specific outcome matches (e.g., cognition and executive capacity) and general outcome matches (e.g., mobility and physical function) were identified within each matched COS set to report the number and percent of distinct outcomes recommended for both settings.

**Results:**

Eighteen matched sets were identified. The median (IQR) number of distinct outcomes recommended for both settings was 6 (4, 8) and the median (IQR) percent of all distinct outcomes that were recommended for both settings, of those included across both settings, was 20 percent (12%, 33%), ranging from nine percent for stroke rehabilitation to 50 percent for psoriatic arthritis. Variation due to potential factors such as outcome granularity, number of matched COS per condition, and scope discrepancies will be discussed. Case studies will be presented for localized and advanced prostate cancer, which demonstrated high levels of overlap.

**Conclusions:**

Within a given condition, there appears to be a subset of outcomes relevant to both research and care, but the degree of overlap varies by disease area. Capturing these overlapping subsets of outcomes within routine data collection could provide a starting point to support generation of real-world evidence, thereby ensuring focus on outcomes of crucial importance to key stakeholders.

